# Adiponectin inhibits LPS-induced nucleus pulposus cell pyroptosis through the miR-135a-5p/TXNIP signaling pathway

**DOI:** 10.18632/aging.205226

**Published:** 2023-12-02

**Authors:** Shuang Wu, Shida Liu, Rui Huang, Youbing Zhou, Yongcheng Zou, Wei Yang, Jian Zhang

**Affiliations:** 1 The First Affiliated Hospital, Orthopedic Center, Hengyang Medical School, University of South China, Hengyang 421001, Hunan, China

**Keywords:** IDD, APN, miR-135a-5p, TXNIP, pyroptosis

## Abstract

Pyroptosis, a newly discovered programmed cell death process, is characterized by NLRP3 inflammasome activation and pro-inflammatory mediator release. Nucleus pulposus (NP) cell pyroptosis is an important cause of intervertebral disc degeneration (IDD). Adiponectin (APN) is an adipokine and has an anti-inflammatory effect. However, whether and how APN protects against NP cell pyroptosis remains unexplored. Our results showed that human degenerated NP tissue displayed a significant increase in the protein levels of NLRP3, caspase-1 and GSDMD-N. APN expression was down-regulated in human degenerated NP tissue and NP cells challenged with lipopolysaccharide (LPS). Lentivirus-mediated overexpression of APN increased miR-135a-5p levels, decreased thioredoxin-interacting protein (TXNIP) expression and its interaction with NLRP3, and inhibited pyroptosis in human NP cells stimulated with LPS. TXNIP was identified as a direct target of miR-135a-5p. The inhibitory effects of APN on pyroptosis were reversed by pretreatment with miR-135a-5p inhibitor or lentiviral vector expressing TXNIP in LPS-treated human NP cells. In summary, these data suggest that APN restrains LPS-induced pyroptosis through the miR-135a-5p/TXNIP signaling pathway in human NP cells. Increasing APN levels could be a new approach to retard IDD.

## INTRODUCTION

As a global challenge, low back pain (LBP) has a considerable effect on economy and society. LBP is predominantly caused by intervertebral disc degeneration (IDD). Cell death and inflammatory response are regarded as two critical pathological mechanisms for IDD [1, 2]. Increased cell death is frequently observed in human degenerated intervertebral disc (IVD) samples and animal models [3, 4]. Pyroptosis is an inflammatory cell death manner. In this process, interleukin (IL)-1β and IL-18 are abundantly released. Nucleus pulposus (NP), the major structural component, is a gelatinous matrix. NP cell pyroptosis has been thought to be a critical contributor to IDD [5, 6].

Adiponectin (APN) is an adipokine with an anti-inflammatory effect [7]. APN is down-regulated in human degenerated IVD, and its levels are negatively correlated with degeneration degree [8]. A strong association exists between circulating APN levels and lumbar disc degeneration [9]. Treatment with APN inhibits NLRP3 inflammasome activation and prevents human aortic epithelial cells from lipopolysaccharide (LPS)-induced pyroptosis [10]. APN also suppresses LPS-induced pyroptosis of vascular smooth muscle cells [11]. However, whether APN can modulate NP cell pyroptosis is unclear.

Dysregulation of miRNAs is known to be involved in IDD progression [12–14]. miR-135a-5p is a pleiotropic miRNA. Activation of farnesoid X receptor alleviates vascular inflammation by increasing miR-135a-5p levels in rats with chronic kidney disease [15]. Thioredoxin-interacting protein (TXNIP) can combine with NLRP3 to induce pyroptosis [16, 17]. Administration of morin diminishes NP cell pyroptosis and mitigates IDD in mice by inhibiting the TXNIP/NLRP3 signaling pathway [18]. Conversely, propionibacterium acnes induces NP cell pyroptosis and promotes the degeneration of IVD in a rabbit model of IDD by activating this pathway [19]. However, whether the miR-135a-5p/TXNIP signaling cascade is associated with APN-regulated NP cell pyroptosis is still elusive. The objective of this research was to explore whether APN can affect NP cell pyroptosis.

## MATERIALS AND METHODS

### Cells, reagents and antibodies

We obtained 293T cells from American Type Culture Collection (Rockville, MD, USA). Lentiviral vector expressing APN or TXNIP was synthesized by Genechem (Shanghai, China). Dulbecco's Modified Eagle Medium (DMEM)/F12 (Gibco, Grand Island, NY, US), Hoechst 33342/propidium iodide (PI) double stain kit (Solarbio, Beijing, China), Lipofectamine 3000 (Invitrogen, Carlsbad, CA, USA), miR-135a-5p mimic/inhibitor (Ribobio, Guangzhou, China), dual-luciferase reporter assay system (Promega, Madison, WI, USA), lactate dehydrogenase (LDH) assay kit (Beyotime, Shanghai, China), enzyme-linked immunosorbent assay (ELISA) kits (R&D systems, Minneapolis, MN, USA), polyvinylidene difluoride (PVDF) membranes (Millipore, Billerica, MA, USA), and horseradish peroxidase-conjugated goat anti-rabbit IgG (Proteintech, Chicago, IL, USA) were obtained as indicated. Fetal bovine serum (FBS), trypsin, type II collagenase and LPS were purchased from Sigma-Aldrich (St. Louis, MO, USA). Rabbit antibodies against APN, TXNIP, NLRP3, caspase-1, Gasdermin N-terminal fragment (GSDMD-N), and β-actin were supplied by Abcam (Cambridge, UK).

### NP tissue sample collection

The patients with lumbar vertebral fracture (LVF) or IDD were collected from the First Affiliated Hospital of University of South China. Information regarding these patients is listed in Supplementary Table 1. NP tissue was carefully isolated after surgery.

### Cell culture and transfection

After washing with phosphate buffered saline (PBS), normal NP tissue were cut into pieces, digested, and then centrifuged at 600 g to collect cells. The isolated NP cells were cultured and passaged. We selected the second-generation cells to perform the *in vitro* experiments. Cells were treated with 1 μg/mL of LPS to induce pyroptosis. Twelve h later, cells were transfected with 100 MOI of lentiviral vector expressing APN or empty vector. After 24 h, the transfection effect was evaluated by detecting APN protein levels.

For overexpression of TXNIP, human NP cells were transduced with 100 MOI of TXNIP-expressing lentivirus or empty vector. To overexpress or silence miR-135a-5p, human NP cells were transfected with miR-135a-5p mimic or its inhibitor (40 nM) at the aid of Lipofectamine 3000. Following 24 h of treatment, TXNIP protein and miR-135a-5p expression was measured for determination of transfection effect.

### Bioinformatics analysis and luciferase reporter detection

The interaction of miR-135a-5p with TXNIP 3′UTR was analyzed by three online databases: TargetScan, miRDB and RNAhybrid. Next, we assayed the luciferase activity according to a previously reported method [20]. Briefly, TXNIP-WT and TXNIP-Mut plasmids were constructed. By using Lipofectamine 3000, these plasmids were cotransfected into 293T cells with miR-135a-5p mimic. Following 24 h, luciferase activity was measured.

### Hoechst 33342/PI fluorescence staining

At the end of treatment, human NP cells were harvested. Hoechst 33342 solution (5 μL) was added to cells. After 20 min of staining, 5 μL of PI was added to cells. Fifteen min later, cells were photographed.

### LDH detection

The cell culture supernatants were collected. After centrifugation, supernatants were incubated with LDH test working solution (60 μL) for 30 min, followed by measurement of absorbance at 490 nm using spectrophotometric microplate reader [21].

### ELISA

The secretion of IL-1β and IL-18 was assessed by the ELISA kits.

### Co-immunoprecipitation (Co-IP) assay

Cells were lysed and then incubated with anti-TXNIP or anti-NLRP3 antibodies overnight. Dynabeads protein G was added. After washing, immunoprecipitates were eluted at 60° C for 5 min with 40 mL of 1 × SDS sample buffer, followed by immunoblotting analysis of TXNIP and NLRP3.

### qRT-PCR

For extracting total RNA in tissue and cells, the TRIzol kit was utilized. After conversion to cDNA, qRT-PCR was carried out and target gene expression was quantified using GAPDH or U6 as internal controls. The primer sequences were as follows: APN (F: 5′-TTGGTCCTA AGGGAGACATCG-3′, R: 5′-CACACTGAATGCTGAGCGGTA-3′), TXNIP (F: 5′-C AGAAGCTCCTCCCTGCTATATG-3′, R: 5′-GATGCAGGGATCCACCTCAG-3′), GAPDH (F: 5′-TGTGGGCATCAATGGATTTGG-3′, R: 5′-ACACCATGTATTCCGGGTCAA T-3′), miR-135a-5p (F: 5′-CTCCTAGGTATGGCTTTTTATTC-3′, R: 5′-TCAACTG GTGTCGTGGAGTC-3′), and U6 (F: 5′-AGTAAGCCCTTGCTGTCAGTG-3′, R: 5′-CCTGGGTCTGATAATGCTGGG-3*′*).

### Western blot

The RIPA buffer was used to isolate total proteins from tissue and cells, which was accompanied by protein concentration detection. Subsequently, SDS-PAGE was employed to separate proteins, which were diverted to PVDF membranes. Following blockade, the PVDF membranes were incubated with the primary antibodies and then secondary antibody. Immunoreactive bands were developed using BeyoECL Plus kit (Beyotime).

### Statistical analyses

The experimental data are expressed as the mean ± standard deviation from at least 3 separate experiments. Differences among groups were analyzed by Student’s *t*-test or one-way ANOVA followed by Tukey’s multiple comparison test. The statistical analyses were conducted using GraphPad Prism 8.0 software. *P* < 0.05 was thought to have statistical significance.

## RESULTS

### APN is down-regulated in degenerated NP tissue and LPS-stimulated NP cellss

**Figure 1:**
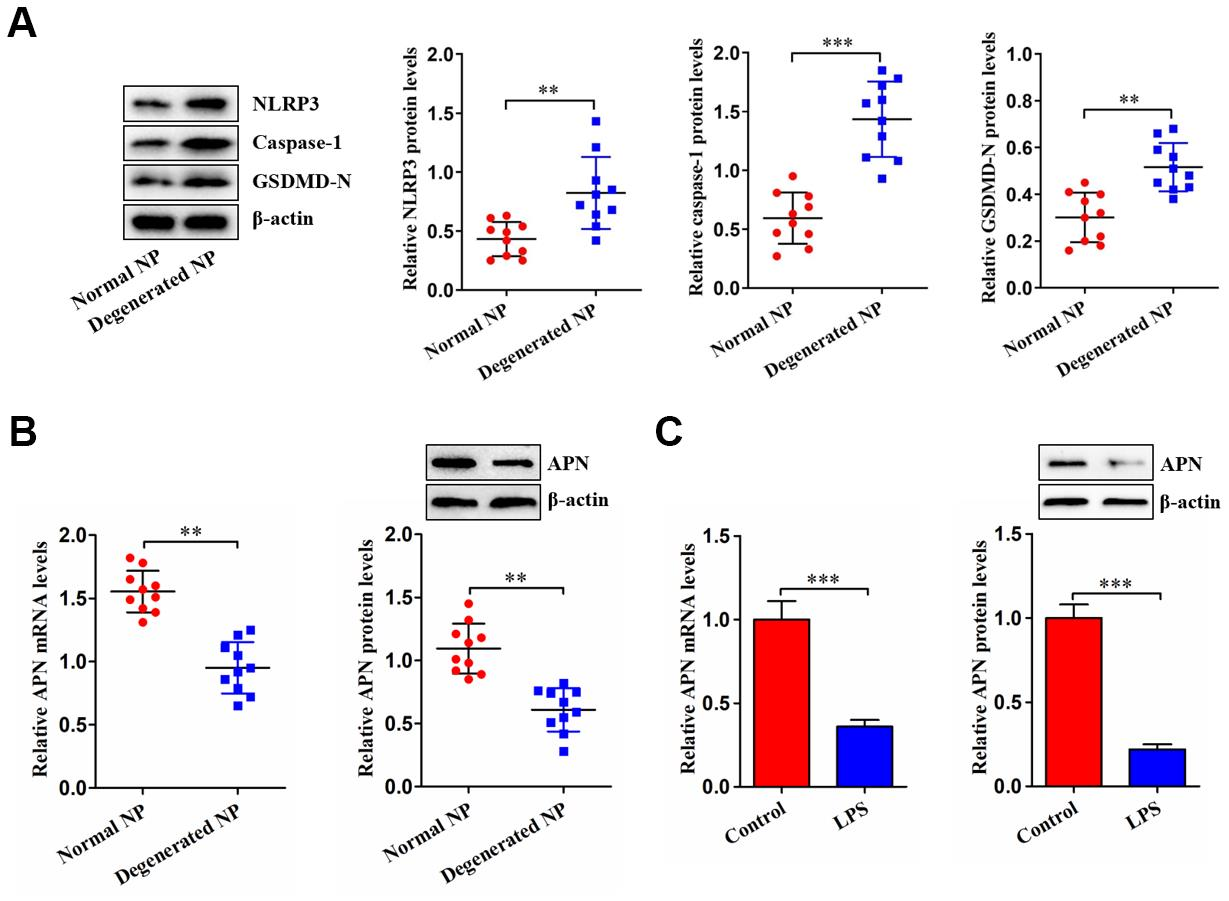
**Decreased APN expression in degenerated NP tissue and LPS-treated human NP cells.** (**A**) Western blot was used to determine the protein levels of NLRP3, caspase-1 and GSDMD-N in normal and degenerated human NP tissue samples (*n*=10). (**B**) The qRT-PCR and western blot analyses of APN expression in normal and degenerated human NP tissue samples (*n*=10). (**C**) Human NP cells were treated with PBS or LPS for 12 h, followed by measurement of APN expression using qRT-PCR and western blot (*n*=3). Data are expressed as mean ± SD. **P < 0.01, ***P < 0.001.

NP tissue was isolated from LVF and IDD patients. Figure 1A shows increased NLRP3, caspase-1 and GSDMD-N expression in human degenerated NP tissue, indicating that NP cell pyroptosis is increased during IDD. We next measured APN expression in NP tissue as well as LPS-stimulated NP cells. Decreased APN expression was observed in degenerated NP tissue (Figure 1B). Consistently, stimulation of human NP cells with LPS dramatically attenuated APN levels (Figure 1C). Thus, APN may be involved in IDD progression.

### APN inhibits LPS-induced pyroptosis in human NP cells

**Figure 2:**
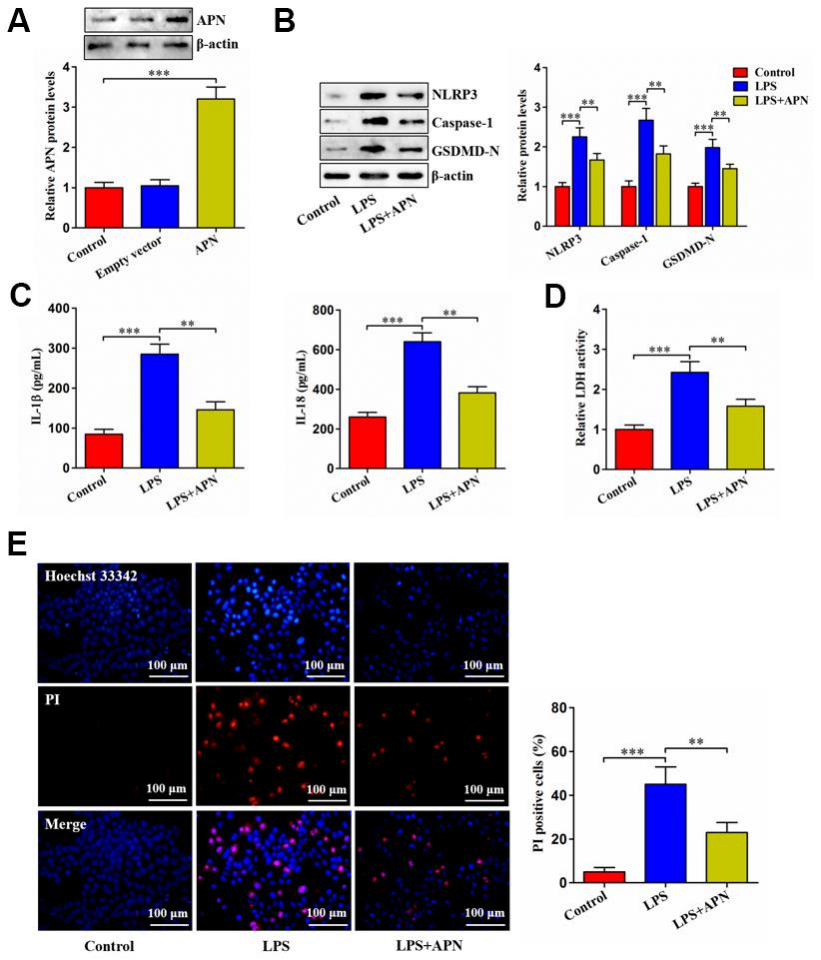
**Effects of APN on LPS-induced human NP cell pyroptosis.** (**A**) Human NP cells were incubated with PBS, empty vector or lentiviral vector expressing APN for 24 h. Protein samples were immunoblotted with antibodies against APN or β-actin. (**B**–**E**) Human NP cells were treated with PBS or LPS for 12 h, followed by transduction with or without APN-expressing lentivirus for 24 h. (**B**) The western blot analysis of NLRP3, caspase-1 and GSDMD-N protein expression. (**C**) ELISA was used to determine IL-1β and IL-18 levels in the cell culture supernatants. (**D**) Detection of LDH activity in the cell culture supernatants using an LDH assay kit. (**E**) Representative images of fluorescent staining with Hoechst 33342 (blue) and PI (red) and quantitative analysis of PI positive cells. All data are presented as mean ± SD from three independent experiments. **P < 0.01, ***P < 0.001.

APN overexpression led to a marked elevation of APN protein levels, as evidenced by western blot (Figure 2A). LPS stimulation dramatically up-regulated NLRP3, caspase-1 and GSDMD-N expression, while this up-regulation was alleviated by APN overexpression (Figure 2B). Accordingly, APN overexpression reversed LPS-induced enhancement of IL-1β and IL-18 secretion (Figure 2C). Consistently, increased LDH release by LPS was alleviated by APN overexpression (Figure 2D). APN overexpression also reduced pyroptotic cell death caused by LPS (Figure 2E). To summarize, APN contributes to inhibition of LPS-induced human NP cell pyroptosis.

### APN decreases TXNIP expression and inhibits its interaction with NLRP3

**Figure 3:**
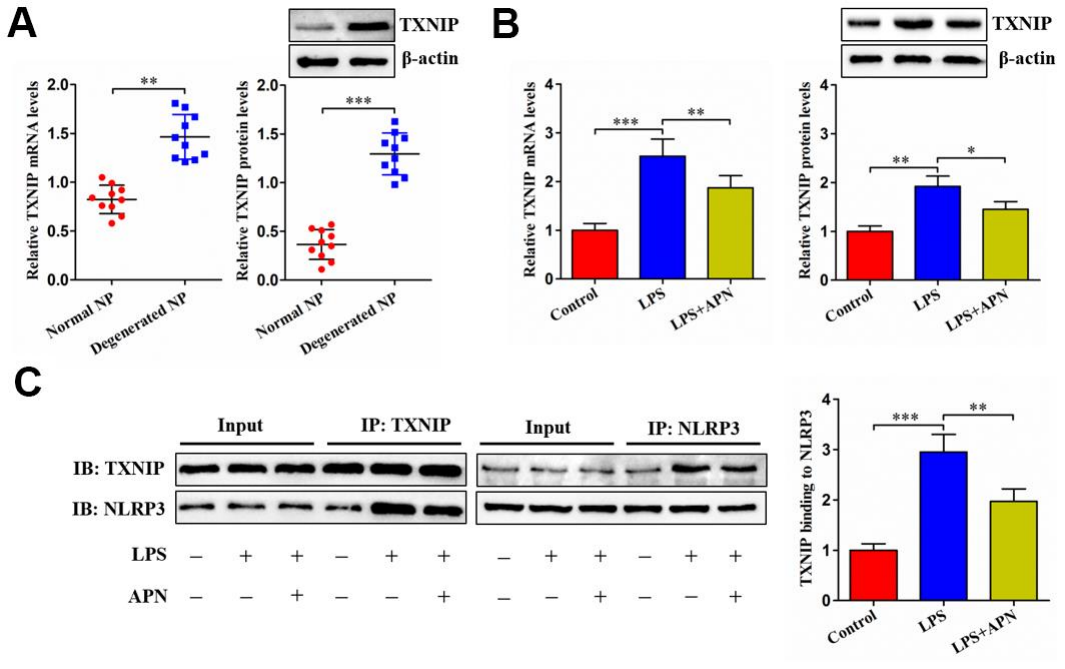
**Effects of APN on TXNIP expression and its interaction with NLRP3.** (**A**) TXNIP expression was assayed by qRT-PCR and western blot in normal and degenerated human NP tissue samples (*n*=10). (**B**, **C**) After 12 h of treatment with PBS or LPS, human NP cells were transfected with or without lentiviral vector expressing APN for 24 h (*n*=3). (**B**) TXNIP expression was evaluated by qRT-PCR and western blot. (**C**) Co-IP analysis of TXNIP interaction with NLRP3. All results are expressed as the mean ± SD. *P < 0.05, **P < 0.01, ***P < 0.001.

Upon binding to NLRP3, TXNIP can induce cell pyroptosis. We inferred that APN inhibits LPS-induced human NP cell pyroptosis possibly through TXNIP. The expression of TXNIP was first detected in NP tissue. As anticipated, TXNIP was highly expressed in degenerated NP tissue samples (Figure 3A). Then, we transfected APN-expressing lentivirus into LPS-treated human NP cells. LPS stimulation dramatically elevated TXNIP mRNA and protein levels, which was alleviated by APN overexpression (Figure 3B). The Co-IP experiment further revealed that APN overexpression reduced the interaction between TXNIP and NLRP3 (Figure 3C). These data indicate that APN down-regulates TXNIP expression and suppresses the binding of TXNIP to NLRP3 in human NP cells stimulated with LPS.

### TXNIP is involved in APN-induced inhibition of human NP cell pyroptosis

**Figure 4:**
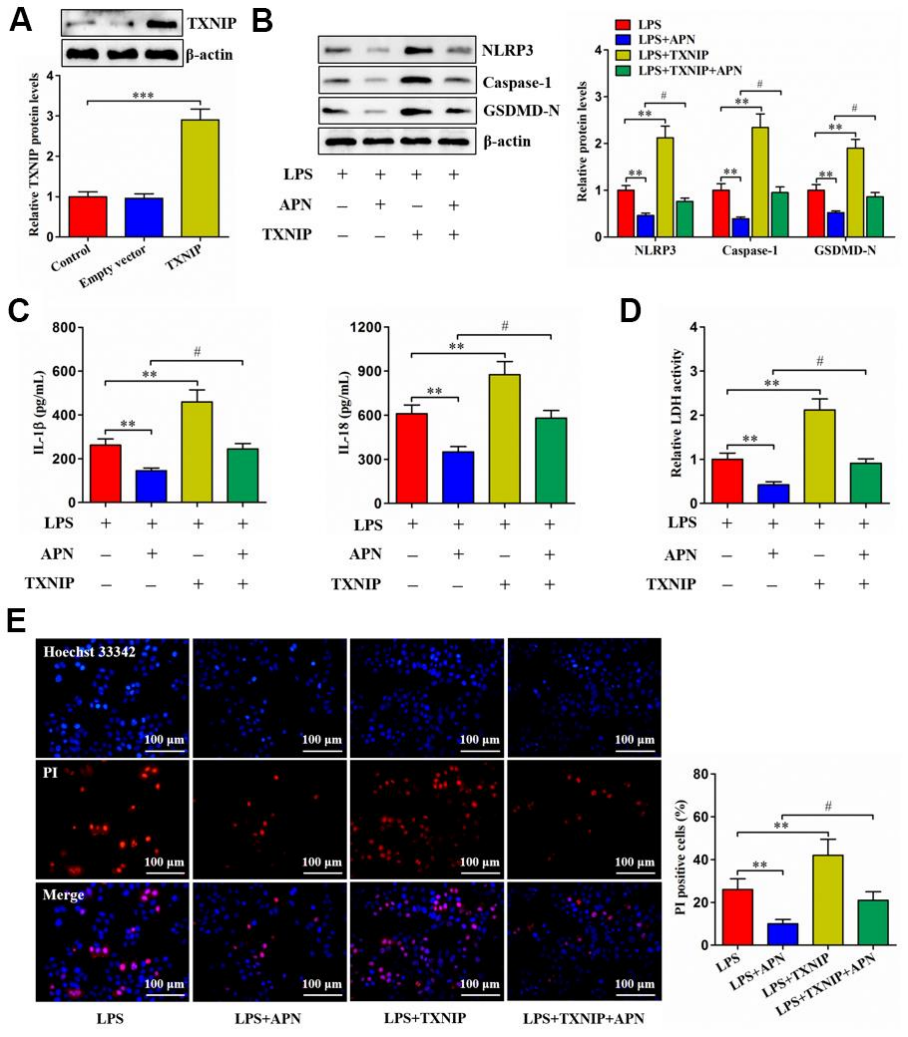
**Involvement of TXNIP in APN-inhibited human NP cell pyroptosis.** (**A**) Human NP cells were treated with PBS, empty vector or lentiviral vector expressing TXNIP for 24 h, followed by western blot analysis of TXNIP protein. (**B**–**E**) Human NP cells were transfected with APN and/or TXNIP by lentiviral vector in the presence of LPS. (**B**) Cell lysates were immunoblotted with indicated antibodies. (**C**) The levels of IL-1β and IL-18 were detected using ELISA in the cell culture supernatants. (**D**) The LDH assay kit was employed to measure LDH activity in the cell culture supernatants. (**E**) Representative images of fluorescent staining with Hoechst 33342 (blue) and PI (red). PI positive cells were quantified. Data shown are mean ± SD from three independent experiments. *P < 0.05, **P < 0.01, ***P < 0.001.

Given the above studies have identified APN as a negative regulator of TXNIP, we overexpressed TXNIP through a lentiviral vector in human NP cells (Figure 4A). LPS-treated human NP cells were then transduced with lentiviral vector expressing TXNIP, followed by treatment with lentiviral vector expressing APN. TXNIP overexpression reduced the influence of APN on pyroptosis-related protein expression (Figure 4B). Additionally, APN overexpression reduced extracellular IL-1β, IL-18 and LDH contents as well as suppressed pyroptotic cell death, both of which were reversed by pretreatment with lentiviral vector expressing TXNIP (Figure 4C–4E). Thus, TXNIP is required for the prevention of human NP cell pyroptosis induced by APN.

### MiR-135a-5p directly targets TXNIP

**Figure 5:**
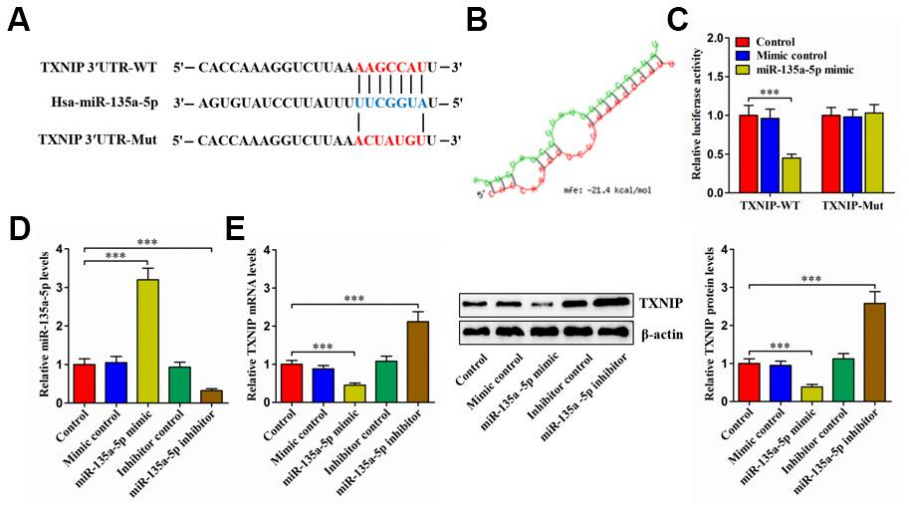
**TXNIP is a direct target of miR-135a-5p.** (**A**) Schematic of miR-135a-5p binding site in the 3′UTR of TXNIP mRNA and corresponding mutation. (**B**) Free energy score predicted by the RNAhybrid database. (**C**) The luciferase reporter plasmids (TXNIP-WT and TXNIP-Mut) were co-transfected into 293T cells with miR-135a-5p mimic or mimic control for 24 h, followed by measurement of luciferase activity. (**D**, **E**) Human NP cells were transfected with miR-135a-5p mimic/inhibitor or their negative controls for 24 h. (**D**) The qRT-PCR analysis of miR-135a-5p expression. (**E**) Detection of TXNIP expression using qRT-PCR and western blot. Data represent the mean ± SD from three independent experiments. ***P < 0.001.

Bioinformatics analyses revealed that miR-135a-5p could directly interact with TXNIP 3′UTR (Figure 5A), with lower free energy score (Figure 5B). Moreover, transfection of 293T cells with miR-135a-5p mimic obviously diminished the luciferase activity of TXNIP-WT (Figure 5C). Subsequently, we overexpressed or silenced miR-135a-5p in human NP cells (Figure 5D). Overexpression of miR-135a-5p markedly diminished TXNIP levels, and an opposite effect appeared after silencing of this miRNA (Figure 5E). Collectively, the above results reveal that miR-135a-5p can directly target TXNIP.

### APN down-regulates TXNIP expression and inhibits human NP cell pyroptosis through miR-135a-5p

**Figure 6:**
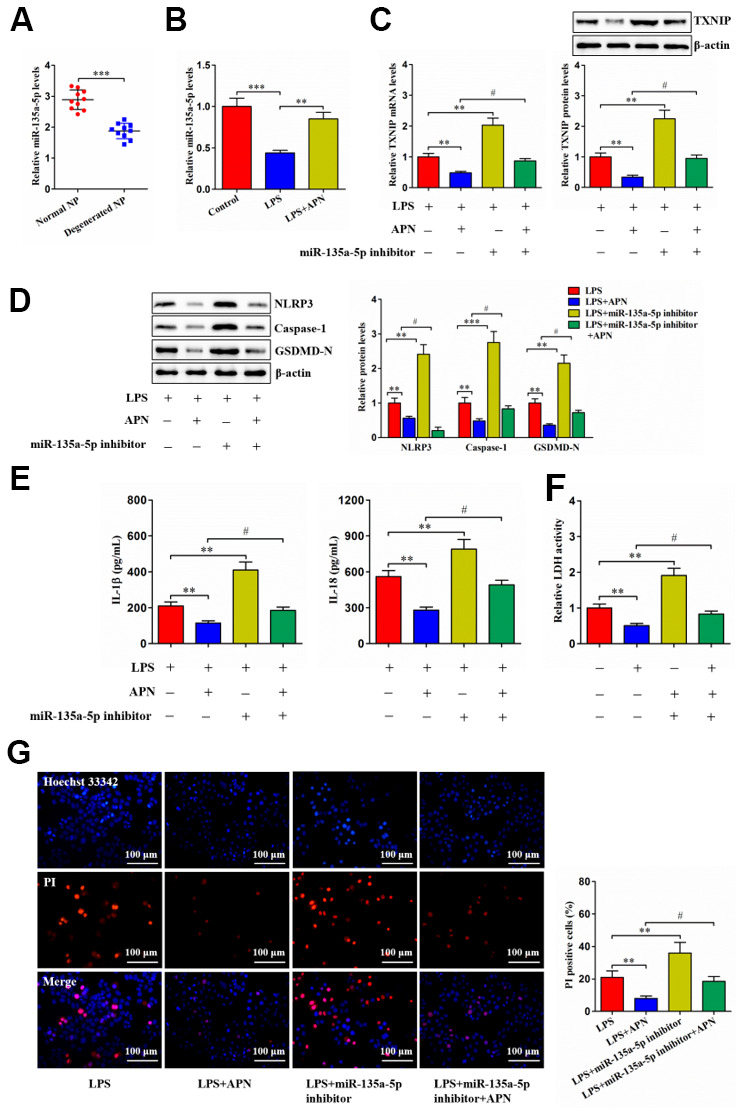
**APN suppresses human NP cell pyroptosis by up-regulating miR-135a-5p expression.** (**A**) Detection of miR-135a-5p expression in normal and degenerated human NP tissue samples using qRT-PCR (*n*=10). (**B**) Human NP cells were treated with PBS or LPS for 12 h and then transfected with or without lentiviral vector overexpressing APN for an additional 24 h. The expression of miR-135a-5p was analyzed by qRT-PCR (*n*=3). (**C**–**G**) LPS-stimulated human NP cells were transfected with or without miR-135a-5p inhibitor for 24 h, followed by transduction with APN-expressing lentivirus for another 24 h (*n*=3). (**C**) TXNIP expression was measured using qRT-PCR and western blot. (**D**) Western blot was applied to determine the protein levels of NLRP3, caspase-1 and GSDMD-N. (**E**) Detection of IL-1β and IL-18 levels in the cell culture supernatants using ELISA. (**F**) The LDH assay kit was utilized to detect LDH activity in the cell culture supernatants. (**G**) Representative images of fluorescent staining with Hoechst 33342 (blue) and PI (red) and quantitative analysis of PI positive cells. The results are shown as the mean ± SD. **P < 0.01, ***P < 0.001, ^#^*P* < 0.05.

For validating whether APN-regulated human NP cell pyroptosis is mediated by miR-135a-5p, we first detected its expression using qRT-PCR. Contrary to TXNIP, decreased miR-135a-5p amount was seen in human degenerated NP tissue samples (Figure 6A). LPS also reduced miR-135a-5p levels within human NP cells, while its reduction was suppressed after APN overexpression (Figure 6B). Next, human NP cells were subjected to miR-135a-5p inhibitor and/or APN-expressing lentiviral vector treatment in the presence of LPS. APN overexpression decreased TXNIP expression, which was abolished after miR-135a-5p inhibition (Figure 6C). APN-mediated down-regulation of pyroptosis-related protein expression was abrogated by miR-135a-5p knockdown (Figure 6D). Also, APN overexpression reduced extracellular IL-1β, IL-18 and LDH contents and pyroptotic cell death, which was reversed upon miR-135a-5p prevention (Figure 6E–6G). Taken together, APN down-regulates TXNIP expression and restrains LPS-induced human NP cell pyroptosis by enhancing miR-135a-5p levels.

## DISCUSSION

APN is produced predominantly by adipose tissue. Notably, skeletal muscle and cardiomyocytes can synthesize APN as well [22, 23]. NP is a major structure component within the IVD tissue and has the ability to generate APN. A recent study demonstrated that human degenerated IVD tissue exhibit a significant reduction in APN expression, and that treatment with APN decreases TNF-α production in degenerated NP cells [8]. Similar to this report, we observed decreased APN expression in degenerated NP tissue. Exposure of human NP cells to LPS could also inhibit APN expression. These findings suggest that decreased APN levels might have a causative role in IDD.

Cell pyroptosis is characterized by membrane pore formation and pro-inflammatory mediator release [24]. NP cell pyroptosis not only triggers inflammatory response within the IVD, but also promotes extracellular matrix degradation [25]. Prevention of NP cell pyroptosis ameliorates IDD in animal models [18]. APN was shown to suppress pyroptosis in aortic epithelial cells [10], vascular smooth muscle cells [11], and hepatocytes [26]. Similarly, we found that lentiviral vector expressing APN diminished pyroptosis-related protein, IL-1β, IL-18 and LDH levels as well as inhibited death occurrence in human NP cells challenged with LPS. Thus, APN plays a protective role in NP cell pyroptosis.

TXNIP is a negative regulator of thioredoxin. In addition to involving oxidative stress, TXNIP serves as a binding partner to NLRP3 [27, 28]. Recently, Yu et al. reported that TXNIP is up-regulated in human degenerated NP tissue samples [29]. Overexpression of TXNIP promotes NP cell pyroptosis and accelerate mouse IVD degeneration by binding to NLRP3; however, an opposite effect is observed when TXNIP is knocked down [18]. In this study, incremental TXNIP expression was seen in human degenerated NP tissue. LPS stimulation also up-regulated TXNIP expression in human NP cells. Importantly, overexpression of APN decreased TXNIP levels and inhibited its interaction with NLRP3 in human NP cells challenged with LPS. APN-induced reduction of NP cell pyroptosis was also alleviated by TXNIP overexpression. Therefore, suppression of NP cell pyroptosis by APN requires TXNIP.

MiRNAs exert biological functions via silencing their target genes [30]. Dysregulated miRNAs take part in inflammatory diseases and IDD [31, 32]. In PC12 cells treated with oxygen-glucose deprivation/reoxygenation, lncRNA SOX2 overlapping transcript promotes the production of pro-inflammatory mediators by sponging miR-135a-5p [33]. Additionally, transfection of HASMCs with miR-135a-5p mimic was shown to attenuate inflammatory cytokine levels [15]. Our present study revealed that miR-135a-5p targeted TXNIP. Importantly, APN-induced inhibition of TXNIP expression and NP cell pyroptosis was attenuated after miR-135a-5p inhibitor transfection. Thus, our data imply that APN-regulated TXNIP expression and NP cell pyroptosis is mediated, at least in part, by miR-135a-5p.

Although this study has clearly demonstrated that APN is protective against LPS-induced human NP cell pyroptosis, there are several limitations in our research. Oxidative stress is known to promote IDD progression besides inflammation [34, 35]. TXNIP also plays a vital role in mediating oxidative stress [36]. Whether APN can affect oxidative stress in NP cells needs to be determined in future research. There is a lack of more immunostaining test for pyroptosis markers, such as caspase-1, to further confirm the occurrence of cell pyroptosis in NP tissue samples and cultured cells. In addition, we do not observe the impact of APN on IDD in animal models.

**Figure 7:**
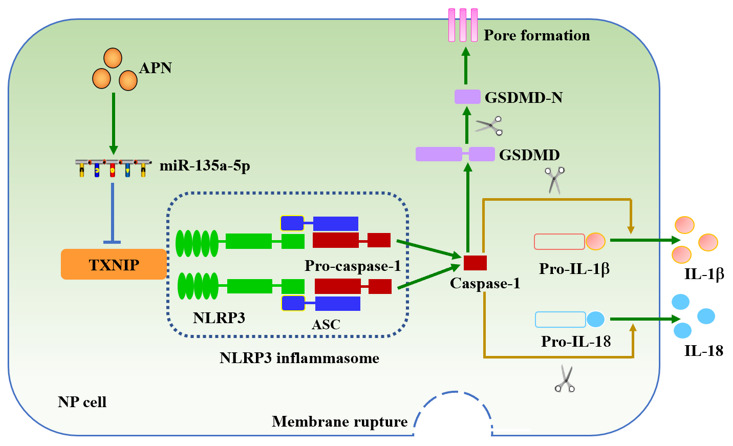
**Schematic diagram of APN-induced prevention of human NP cell pyroptosis.** APN increases miR-135a-5p levels and then down-regulates TXNIP expression, leading to decreased interaction between TXNIP and NLRP3 in human NP cells stimulated with LPS. Inactivation of the NLRP3 inflammasome inhibits membrane pore formation and subsequent release of IL-1β and IL-18.

In conclusion, APN exerts an inhibitory effect on NP cell pyroptosis, which is dependent on the miR-135a-5p/TXNIP signaling pathway (Figure 7). These findings deepen our understanding for the role of APN in the pathogenesis of IDD. Targeting APN could be a potential strategy to prevent and treat IDD.

## Supplementary Material

Supplementary Table 1
