# Supplementary material for: Adiponectin inhibits LPS-induced nucleus pulposus cell pyroptosis through the miR-135a-5p/TXNIP signaling pathway
Source: Aging (Albany NY). 2023 Dec 2;15(23):13680–92. doi: 10.18632/aging.205226 (PMC10756118; doi:10.18632/aging.205226)
Supplement: Supplementary Table 1 [file aging-15-205226-s001.pdf]

## SUPPLEMENTARY TABLE

**Supplementary Table 1. Summary of clinical and demographic features of patients.**

| Subject number | Gender | Age (years) | Level | Pfirrmann grading |
|---|---|---|---|---|
| Normal control group | | | | |
| 1 | Male | 18 | L1/2 | II |
| 2 | Male | 16 | L4/5 | II |
| 3 | Female | 20 | L2/3 | II |
| 4 | Male | 21 | L2/3 | II |
| 5 | Male | 19 | L4/5 | II |
| 6 | Male | 24 | L3/4 | II |
| 7 | Female | 22 | L3/4 | II |
| 8 | Male | 23 | L4/5 | II |
| 9 | Female | 25 | L2/3 | II |
| 10 | Female | 18 | L4/5 | II |
| IDD group | | | | |
| 1 | Male | 57 | L4/5 | IV |
| 2 | Male | 40 | L5/S1 | V |
| 3 | Female | 45 | L4/5 | V |
| 4 | Female | 60 | L4/5 | IV |
| 5 | Male | 55 | L3/4 | IV |
| 6 | Female | 54 | L4/5 | IV |
| 7 | Female | 57 | L5/S1 | V |
| 8 | Male | 48 | L5/S1 | IV |
| 9 | Female | 58 | L3/4 | IV |
| 10 | Male | 51 | L4/5 | V |
